# The effect of androgens on ovarian follicle maturation: Dihydrotestosterone suppress FSH-stimulated granulosa cell proliferation by upregulating PPARγ-dependent PTEN expression.

**DOI:** 10.1038/srep18319

**Published:** 2015-12-17

**Authors:** Mei-Jou Chen, Chia-Hung Chou, Shee-Uan Chen, Wei-Shiung Yang, Yu-Shih Yang, Hong-Nerng Ho

**Affiliations:** 1 Department of Obstetrics and Gynecology, National Taiwan University Hospital and College of Medicine, National Taiwan University, Taipei, Taiwan; 2 Department of Internal Medicine, National Taiwan University Hospital, Taipei, Taiwan; 3 Graduate Institute of Clinical Medicine, College of Medicine, National Taiwan University, Taipei, Taiwan

## Abstract

Intraovarian hyperandrogenism is one of the determining factors of follicular arrest in women with polycystic ovary syndrome (PCOS). Using androgenized rat models, we investigated the effects of androgens on metabolism, as well as on factors involved in follicular arrest and the reduced number of estrus cycles. The dihydrotestosterone (DHT)-treated rats had fewer estrus cycles, higher numbers of large arrested follicles and an increased in body weight gain compared with the dehydroepiandrostenedione (DHEA)- and placebo-treated rats. In cultured rat granulosa cells, DHT suppressed follicle stimulating hormone (FSH)-induced granulosa cell proliferation and increased the accumulation of cells in the G2/M phase. DHT decreased phosphorylated Akt (p-Akt) and cyclin D1 levels through increasing PTEN. DHT-promoted PTEN expression was regulated by peroxisome proliferator-activated receptor gamma (PPARγ) in granulosa cells. Meanwhile, in the large follicles of the DHT-treated rats, the expressions of PPARγ and PTEN were higher, but the expression of p-Akt and proliferating cell nuclear antigen (PCNA) were lower. Conclusively, DHT and DHEA produced differential effects on metabolism in prepubertal female rats like clinical manifestations of women with PCOS. DHT treatment may affect ovarian follicular maturation by altering granulosa cell proliferation through the regulation of enhancing PPARγ dependent PTEN/p-Akt expression in the granulosa cells.

Polycystic ovary syndrome (PCOS) is the most common endocrinopathy among women of reproductive age^1^ and it is characterized by both reproductive and metabolic disorders^2^,^3^. The androgen excess has been suggested as a necessary criterion for the diagnosis of PCOS^4^,^5^.

The presence of either adrenal or ovarian hyperandrogenemia is considered the main characteristic of PCOS and may play a pivotal role in its pathogenesis^6^,^7^,^8^. However, androgens from the adrenal gland or ovaries have been reported to have differential effects on the phenotypes of women with PCOS^6^,^7^,^9^,^10^,^11^. Women with PCOS who have high adrenal androgen levels in the form of dehydroepiandrosterone sulfate (DHEAS), which is mostly converted from dehydroepiandrosterone (DHEA), have been reported to be less obese and have better insulin sensitivity and metabolic profiles than those who have high ovarian androgen levels in the form of testosterone and its irreversible metabolite, dihydrotestosterone (DHT)^9^,^10^,^11^.

The dysfunctional reproductive and metabolic characteristics caused by DHT has been reported in mice^12^,^13^ and rats^14^,^15^ treated postnatally from 19 to 21 days of age (prepubertal) with 90-day continuous-release pellets containing DHT. DHT-treated rodent models exhibit many reproductive and metabolic features similar to those of obese women with PCOS, including irregular estrus cycles, increased numbers of large atretic follicles, increased body weight and visceral fat, dyslipidemia, and aggravated insulin resistance^13^,^14^,^15^. However, little is known about the mechanisms underlying the reduced numbers of estrus cycles and follicle arrest observed in androgenized rats.

Rodent models have been used to assess the effects of DHEA, a weak androgen, since 1962^16^, when DHEA and androstenedione levels were observed to be abnormally increased in the ovaries of women with PCOS^17^. Most of the previous studies of DHEA-treated rodent models^16^,^18^,^19^,^20^,^21^ that exposed to DHEA treatment have assessed short-term treatment over only 2 to 4 weeks. There have been controversial results with regard to body weight changes after DHEA treatment, and there are limited data on the metabolic disturbance induced by DHEA. Such inconclusive results have limited the ability to interpret the relationship between DHEA and metabolic disturbances and to apply the findings to women with PCOS^21^. Though there has been a recent mice study that revealed no PCOS features after long-term treatment of DHEA^12^, however, that might be the result of low DHEA dosage used in that study; furthermore, the long-term DHEA treated rat models is still not available in the literatures.

In this study, we aimed to determine whether the long-term administration of DHEA and DHT lead to different reproductive and metabolic phenotypes in rats, as has been shown in women with PCOS. In addition, we aimed to identify the factors involved in the follicular arrest and reduced estrous cycles observed in androgenized rat models by studying the effect of androgen on the expression of p-Akt and phosphatase and tensin homolog deleted on chromosome 10 (PTEN), which have been reported to be involved in granulosa cell survival^22^,^23^ and follicular development^24^,^25^.

## Material and Methods

### Animals and study procedure

At 21 days of age, immature female Sprague-Dawley rats were randomly divided into three experimental groups (control, DHT, and DHEA) and received subcutaneous implants of 90-day continuous-release pellets (Innovative Research of America, Sarasota, FL, USA) containing 7.5 mg DHT (daily dose, 83 μg), 200 mg DHEA (daily dose, 2.2 mg), or 7.5 mg placebo. The dose of DHT was chosen to mimic the hyperandrogenic state of women with PCOS, whose plasma DHT levels are approximately 1.7-fold higher than those of healthy controls, according to a previous study^14^. The dose of DHEA chosen in this study was approximately 4- to 6- fold higher than the daily supplemental dose of DHEA suggested for young and older women^26^,^27^. A recently reported long-term DHEA treated mice model that used a 7.5mg DHEA 90-day continuous-release pellets revealed no different finding concerning reproduction and body composition in comparison to the placebo groups^12^. Because of the body weight proportionality and concerning of the weak androgenic potency of DHEA, we then chose the daily dose of 2.2 mg per rat. The controls received identical pellets lacking any bioactive ingredients. All the rats were housed two to three per cage under controlled conditions (65 ± 5% humidity, 22 ± 1 °C temperature, and a daily 12-hour light/12-hour dark cycle) and were fed a commercial chow diet and tap water ad libitum. The rats were weighted weekly from 3 weeks of age and were euthanized after the study was completed, at approximately 15–16 weeks of age.

For the experiments that involved measuring food consumption, dietary intake of each cage was recorded weekly by weighing dispensed, remaining and spilled food. All the animal care procedures and experiments were performed according to the Assessment and Accreditation of Laboratory Animal Care-approved guidelines using protocols approved by the Institutional Review Board-Institutional Animal Care and Use Committee of National Taiwan University Hospital. All the experiments were performed using ketamine for anesthesia. Three to four rats per group were used in each experiment, and each experiment was repeated at least three times.

### Blood sampling and assessment of body composition

At the 9-week experimental period (12^th^ week of age), tail blood was collected after an overnight fast to assess the lipid profile, and the sugar, insulin and liver enzyme levels. At the end of the 12- or 13-week experimental period and after an overnight fast, all the rats were anesthetized and killed by cardiac puncture with concurrent blood sampling for analyses of hormonal profiles. All the blood collections at the end of the experiments were performed during the estrus phase, as determined by daily vaginal smears. At the end of the study, the ovaries were excised, weighed, fixed in neutral-buffered 4% formaldehyde for 24 hours, washed with distilled water, dehydrated, and embedded in paraffin. The liver, hind limb muscles (extensor digitorum longus, tibialis anterior, and soleus), subcutaneous fat depots (including the inguinal region), and visceral fat depots (periovarian, mesenteric and retroperitoneal fat) were all dissected and weighed.

### Vaginal smears for estrus phase determination

The stage of the estrous cycle was determined by daily vaginal smears taken 3 weeks before the rats were euthanized. The vaginal smear was performed according to the following procedure: a small amount of a saline solution was inserted into the rat’s vagina with a disposable pipette, removed, placed on a slide and examined under a microscope. The average interestrus interval was defined as the average duration of the dominant presence of characteristic non-nucleated, cornified epithelial cells with a high cell density during the total 3 weeks of experiments for vaginal smears.

### Ovarian histology and antral follicle counts

The ovaries were longitudinally and serially sectioned at 4 μm and mounted on a glass slide; every 5^th^ section (for a total of 5 slides per ovary) was stained with hematoxylin and eosin. The average antral follicle counts of the sections were determined using a conventional birefringence microscope by two observers who were blind to the origin of the sections. Only antral follicles with a well-defined antral cavity and follicular fluid, including small antral, large antral and Graafian follicles, were counted and considered in the final analyses, and only one ovary per rat was analyzed. The mean antral follicle counts were determined under low magnification (40×), and the results are expressed as the average count of five selected fields per slide, which was then averaged again for a total of five slides per ovary.

The follicular size was determined by measuring the largest diameter at magnification of 200× (for small antral follicls), or 100× and 40× (for large antral follicles). The presence of corpora lutea and Graafian follicles was also determined. Based on the mean diameter and morphology of the follicles, a small antral follicle was defined as having more than 6 layers of cuboidal granulosa cells with a well-defined antral cavity less than 500 μm in diameter. A large antral follicle was defined as having a well-defined antral cavity more than 500 μm in diameter. These criteria were modified from previous studies^13^,^28^.

### Antibodies and reagents

Antibodies against p-PTEN (Ser 380/Thr 382/383), PTEN, p-Akt, Akt, proliferating cell nuclear antigen (PCNA), cyclins D1, A, B and E, beta-actin, tubulin, follicle stimulating hormone (FSH) receptor, p53, early growth response protein 1 (Egr-1) and peroxisome proliferator-activated receptor gamma (PPARγ) were purchased from Santa Cruz Biotechnology (Santa Cruz, CA, USA). A TUNEL assay kit was purchased from R&D Systems.

FSH (working in 10 ng/ml), insulin (working in 500 nM), Dehydroepiandrosterone (DHEA) solution (1.0 mg/mL in methanol ), 5α-Dihydrotestosterone (DHT) solution (1.0 mg/mL in methanol), and Bicalutamide (working in 50 μM) were all purchased from Sigma (Sigma, St Louis, MO).

### Immunohistochemistry

Cross-sections of the ovaries were deparaffinized, rehydrated, and microwaved in 0.01 M citrate buffer (pH 6.0) for antigen retrieval. The sections were then blocked with normal serum and incubated with various antibodies, as indicated. The immunoreactivities were visualized using the ABC staining system (Vector Laboratories, Burlington, CA, USA) following the manufacturer’s instructions. The sections were counterstained with Mayer’s hematoxylin. The percentage of granulosa cells with positive staining was counted in a total of 1000 granulosa cells in a large follicle under a microscopy at 400× magnification for 3 successive slides per ovary, and 5 follicles per ovary were counted. A total of 3 ovaries per group and only 1 ovary per rat were collected for immunohistochemistry analysis.

### Granulosa cell isolation and culture

Granulosa cells were isolated from freshly removed ovaries of 3-week-old Sprague-Dawley rats without prior treatment and cultured using previously described methods with modifications^29^,^30^. Briefly, the ovaries were incubated in cold serum-free medium, consisting of 15 mM HEPES (pH 7.4), Dulbecco’s modified Eagle’s medium (DMEM)/F-12, human insulin (2 μg/ml), hydrocortisone (40 ng/ml), and antibiotics. After being incubated in medium containing 0.5 M sucrose and 10 mM EGTA at 37 °C for 30 min, the ovaries were washed in fresh DMEM/F-12, and granulosa cells were removed from the ovaries by puncturing the follicles with a 25-guage hypodermic needle. The collected cells were incubated in DMEM/F12 medium supplemented with 10% FBS (GIBCO BRL, Grand Island, NY, USA) under a humidified atmosphere of 5% CO_2_ at 37 °C. The purity of the granulosa cells was confirmed by immunostaining for FSH receptor (FSHR). Ratio of FSHR positive cells were analyzed by quantifying the FSHR-positive cells by flow cytometry using a FACS scan and the Cell Quest software (Becton Dickinson Immunocytometry Systems, San Jose, CA, USA).

### Methods of assaying plasma hormonal and metabolic profiles

The plasma levels of FSH, luteinizing hormone (LH), estradiol, and progesterone were determined using commercial kits for rat (FSH and LH: Adaltis Italia S.P.A., BO, Italy; estradiol and progesterone: Siemens Healthcare Diagnostics, NY, USA). The plasma concentrations of testosterone, DHEAS, and androstenedione were assayed using a radioimmunoassay (RIA; Diagnostic Systems Laboratories, Webster, Texas, USA). The plasma levels of total cholesterol (TC), high-density lipoprotein cholesterol (HDL-C), low-density lipoprotein cholesterol (LDL-C), triglycerides (TGs), aspartate aminotransferase (AST), alanine aminotransferase (ALT) and glucose were measured using the respective kits and autoanalyzers (HDL-C and LDL-C: Denka Seiken, Japan with the Toshiba TBA-c16000 autoanalyzer, Toshiba, Tokyo, Japan; glucose, TC, TGs, AST and ALT: VITROS Chemistry, NY, USA, using the VITROS 5.1FS analyzer, Ortho-Clinical Diagnostics, Rochester, NY, USA). The serum insulin levels were determined using a microparticle enzyme immunoassay with the AxSYM system (Abbott Laboratories, Dainabot Co., Tokyo, Japan). The levels of adiponectin were determined using enzyme immunoassay (B-Bridge International Inc. CA, USA). All the samples were measured in one assay.

### Flow cytometry

A FACS scanner and Cell Quest software (Becton Dickinson Immunocytometry Systems, San Jose, CA, USA) were applied to determine the cell cycles of the treated cells by quantifying their DNA contents with propidium iodide staining and to determine the purity of granulosa cell by quantifying the FSHR-positive cells with isotype control by using fluorescence intensity detection.

### Cell proliferation and viability determination by MTT assay

Granulosa cells were plated into 96-well microplates at a density of 1 × 10^3^ cells/well for the cell proliferation assay. Briefly, the cells were cultured at 37 °C for the indicated time, and 30 μl of MTT solution (5 mg/mL) was added into each well, then incubated for 4 hours in dark. The formazan grain was then dissolved in DMSO, and the absorbance was measured at 570 nm using an ELISA plate reader.

### Protein extraction and Western blot analysis

Each treatment within each culture was replicated for at least 3 times. The granulosa cells (1 × 10^6^ cells/well) were cultured in 6-well plate. Cells were lysed using a lysis buffer (1% Triton X-100, 150 mM, NaCl, 1 mM EGTA, 1% NP-40, 1 mM NaF, 1 mM Na3VO4, 2 mM phenylmethyl- sulfonylfuoride (PMSF), 1 mg/ml aprotinin and leupeptin in PBS) and centrifuged at 12,000 rpm for 25 min at 4 °C. For ovarian tissue protein extraction, each ovarian tissue was homogenized and extracted in lysis buffer. The cell lysates or tissue lysates were purified and quantified using a Bio-Rad protein assay (Bio-Rad Laboratories, Hercules, CA, USA). A total of 50-μg protein sample was separated using SDS-PAGE, transferred onto a polyvinylidene difluoride (PVDF) membrane, and immunoblotted with various antibodies. Bound antibodies were detected using the appropriate peroxidase-coupled secondary antibodies and an enhanced chemiluminescence detection system (ECL, Boehringer Mannheim, Indianapolis, IN, USA).

### RNA interference

Small interfering RNA duplexes (siRNA) of PTEN gene (SC-61873), p53 gene (SC-45917), Egr-1 gene (SC-270177) and PPARγ gene (SC-43530) were purchased from Santa Cruz Biotechnology. Negative control siRNAs (Invitrogen Corporation, Carlsbad, CA, USA) with sequences with no similarity to any gene product was used as control. Lyophilized siRNA duplex was resuspended in RNase-free water at a concentration of 10 mM with 10 mM Tris–HCl, pH 8.0, 20 mM NaCl, and 1 mM EDTA buffered solution. The siRNAs were incubated for 15 min at room temperature to allow for complex formation between the siRNA and TransFast^TM^ Transfection Reagent (Promega, Southampton, UK). Rat granulosa cells (1 × 10^5^ cells in a 6-cm dish) were transfected with siRNAs in serum-free Opti-MEM (Invitrogen Corporation, Carlsbad, CA, USA) at a concentration of 25 nM by incubation for 1 hour at 37 °C. The culture medium of the cells was then changed, and they were incubated for 24 hours at 37 °C prior to experiments.

### Real-time reverse transcriptase (RT)-polymerase chain reaction (PCR)

For PPARγ, forward: 5′-CAC AAT GCC ATC AGG TTT GG-3′; reverse: 5′-GCT GGT CGA TAT CAC TGG AGA TG-3′. For PTEN, forward : 5′-ACA CCG CCA AAT TTA ACT GC-3′ and reverse : 5′-TAC ACC AGT CCG TCC TTT CC-3′. For β actin, forward: 5′-AGA GGG AAA TCG TGC GTG AC-3′; reverse: 5′-CCA TAG TGA TGA CCT GTC CGT-3′. Total RNA was isolated from rat granulosa cells using Trizol reagent (Gibco BRL, Rockville MD, USA), according to the manufacturer’s instructions. A total of 2 μg RNA was used to synthesize cDNA in a 25 μmL total reaction volume using a reverse transcription kit (Promega). Amplification was followed by melting curve analysis to verify the correctness of the amplicon. The amount of PPARγ or PTEN mRNA was normalized by that of β actin mRNA and are presented in arbitrary units, with 1 U corresponding to the value in cells treated with a vehicle control.

### Statistical analysis

**Figure 1:**
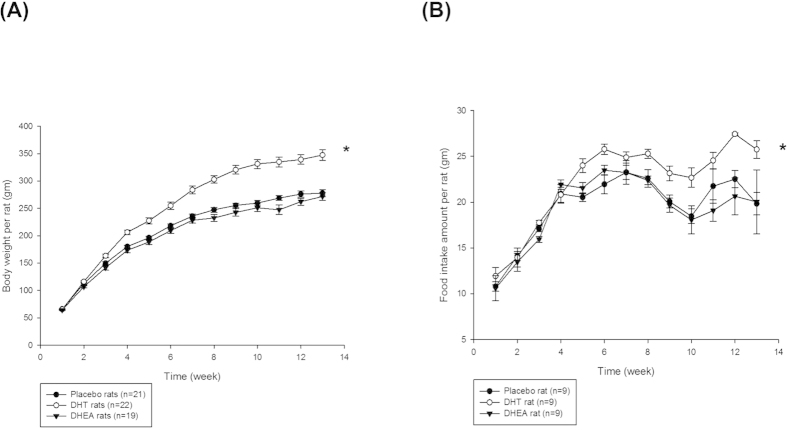
Body weight and food intake in androgenized rats. Female SD rats were randomly divided into three experimental groups (control, DHT, and DHEA) and subcutaneously implanted with 90-day continuous-release pellets containing 7.5 mg DHT (daily dose, 83 μg), 200 mg DHEA (daily dose, 2.2 mg), or 7.5 mg placebo. The data are shown as means ± SD. (**A**) Weekly body weight per rat. Numbers of rats = 22, 19, and 21 for the DHT, DHEA and placebo groups, respectively. (**B**) Weekly food intake amount per rat. Number of rats = 9 in each group. In both (**A**,**B**), the levels of the DHT group were significantly higher than those of the other 2 groups, as determined by repeated measures ANOVA. *P < 0.05.

**Figure 2:**
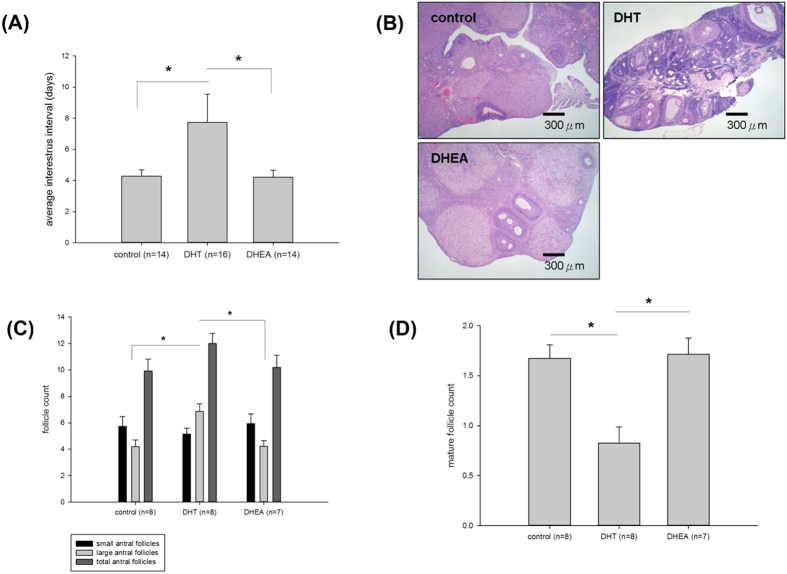
Effects of DHT and DHEA on estrous cycles and follicular maturation. The data are shown as means ± SD. (**A**) The interestrus intervals of the rats were determined by vaginal smears. The interestrus interval of the DHT group was significantly higher than that of the other two groups. Number of rats = 16, 14, and 14 in the DHT, DHEA and placebo groups, respectively. (**B**) At the end of the study, the rat ovaries were fixed for histology. Representative HE staining is shown (40×), bar = 300 μm. (**C**) Quantitative results of total antral follicle count, including small and large antral follicles, among the 3 groups. The large follicle count of the DHT group was significantly higher than that of the other 2 groups. (**D**) Quantitative results of the mature and ovulated follicle count, including the Graafian follicles and corpus lutea, among the 3 groups. The mature and ovulated follicle count of the DHT group was significantly lower than that of the other 2 groups. For the antral follicle measurements in (**C**,**D**), number of rats = 8, 7, and 8 in the DHT, DHEA and placebo groups, respectively. Between-group comparisons are indicated. *P < 0.05.

**Figure 3:**
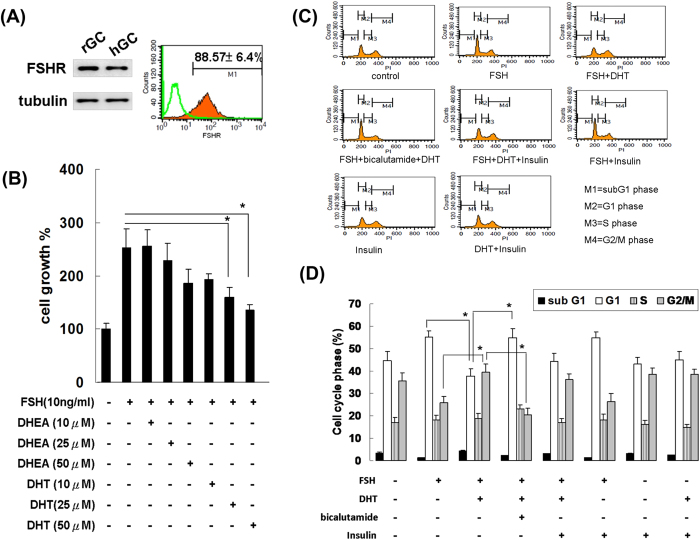
DHT inhibited *in vitro* FSH-induced granulosa cell proliferation through interfering cell cycle. (**A**) Fifty μg of total protein form rat granulosa cells and human granulosa cells (hGC) were used to determine the specificity of the antibody to FSHR of rat granulosa cells (rGC) by immunoblotting (left panel). Ratio of FSHR positive cells were analyzed by quantifying the FSHR-positive cells by flow cytometry using a FACScan and the Cell Quest software. The white histograms are of isotype controls, whereas the orange overlays were of FSHR-positive cells (right panel). (**B**) Rat granulosa cells were treated with FSH and different doses of DHT or DHEA, and after 72 hours, cell growth was determined by the MTT assay. The data are shown as means ± SD of 3 independent experiments. Granulosa cell growth was significantly suppressed by co-treatment with FSH and DHT (exceeding 25 uM) in comparison with FSH treatment only. (**C**) Rat granulosa cells were pretreated with DHT (50 μM), bicalutamide (50 μM), or insulin (500 nM) for one hour prior to FSH (10 ng/ml) treatment. After 24 hours, the cell cycle phase was determined by propidium iodide staining and FACScan analysis. Populations in the subG1, G1, S, and G2/M phases are shaded M1 to M4, respectively. Representative cell cycle histograms of each group are shown. N = 3. (**D**) The percentage of cells in each cell cycle phase was analyzed and quantified by Cell Quest software, and the data represent the mean ± SD of 3 independent experiments. The percentage of cells in the G2/M phase significantly decreased following treatment with FSH. Compared with cells treated with FSH alone, the number of cells in G2/M phase increased significantly following treatment with DHT and FSH. The percentage of cells in G2/M phase significantly decreased following treatment with DHT/FSH/Bicalutamide, in comparison with cells treated with DHT and FSH. Between-group comparisons were performed as indicated *P < 0.05.

**Figure 4:**
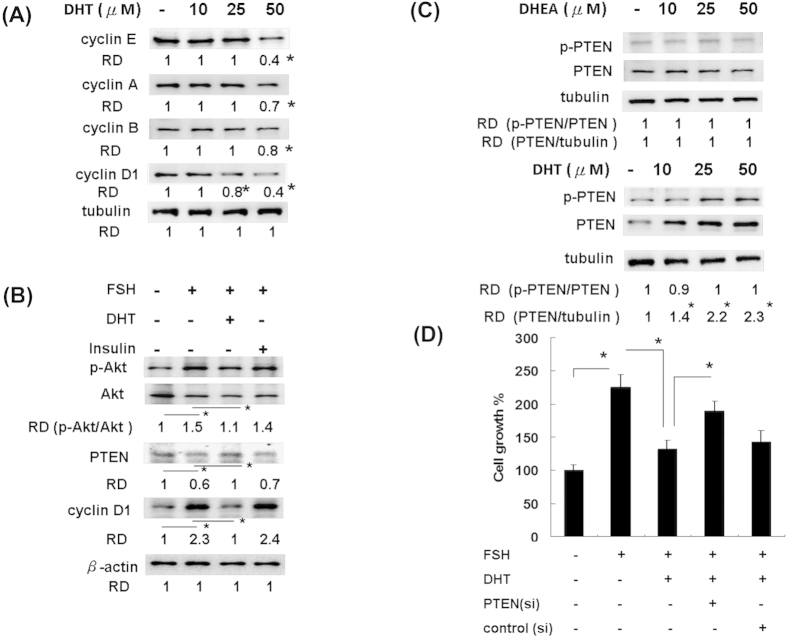
DHT inhibited cell proliferation through regulating p-Akt expression by enhancing PTEN and suppressing resultant cyclin D1 expression. The data are means ± SD. (**A**) Rat granulosa cells were pre-treated with different doses of DHT for 1 hour prior to FSH treatment, and after 24 hours, cell lysates were used to measure the cyclin protein levels by western blot. Expression of tubulin was used as loading control. Representative immunoblottings of protein are shown. N = 3. Relative density (RD) of each lane was determined by ImageJ, lane 1 was defined as 1. Statistical comparisons were done between lane 1 with the other lane. *P < 0.05. (**B**) Rat granulosa cells were pre-treated with DHT (50 μM) or insulin (500 nM) for 1 hour prior to FSH (10 ng/ml) treatment, and after 24 hours, cell lysates were used to measure the indicated protein level by western blot. Expression of β-actin was used as loading control. Representative immunoblottings of protein are shown. N = 3. ImageJ determined RD and lane 1 was defined as 1. Statistical comparisons were done between lane 1 with the other lane. *P < 0.05. (**C**) Rat granulosa cells were pre-treated with DHT or DHEA, and after 24 hours, cell lysates were used to measure the PTEN level by western blot. Expression of tubulin was used as loading control. Representative immunoblottings of protein are shown. N = 3. ImageJ determined RD and lane 1 was defined as 1. Statistical comparisons were done between lane 1 with the other lane. *P < 0.05. (**D**) Rat granulosa cells were pretreated with PTEN siRNA(si) or control siRNA(25 μM) for 24 hours and following with or without FSH(10ng/ml) and DHT(50 μM) treatment. After 3 days, cell growth was determined by the MTT assay, N=3. Between-group comparisons were performed as indicated *P < 0.05.

**Figure 5:**
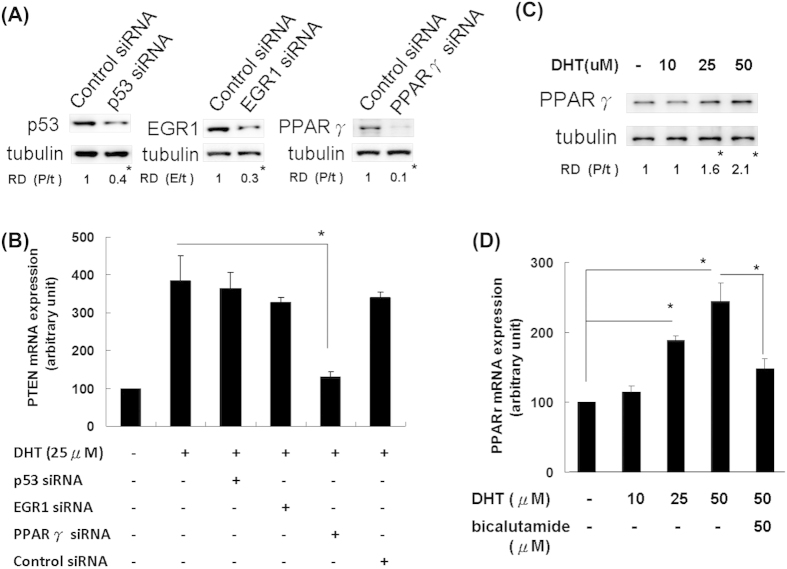
DHT promoted PTEN expression by enhancing PPARγ expression in ovarian granulosa cells. (**A**) Rat granulosa cells were pretreated with siRNAs of p53, Egr-1, and PPARγ for 24 hours, then the protein levels of p53, Egr-1, PPARγ were determined by western blot and significantly decreased after corresponding siRNAs treatment. *P < 0.05. (**B**) Rat granulosa cells were pretreated with siRNAs of p53, Egr-1 and PPARγ respecitvely for 24 hours prior DHT (25uM) treatment for another 24 hours, then PTEN mRNA expression was determined by Q-RT-PCR. PPARγ siRNA significantly suppressed the DHT-upregulated PTEN expression in ovarian granulosa cells. *P < 0.05. (**C**) PPARγ protein levels were determined by western blot after treatment of different doses of DHT for 24 hours in rat granulosa cells. The levels of PPARγ were significantly increased with incremental dosage of DHT treatment. Statistical comparisons were done between lane 1 with the other lane, *P < 0.05, N = 3. (**D**) PPARγ mRNA levels were determine by Q-RT-PCR after treatment of different doses of DHT for 24 hours in rat granulosa cells. The mRNA levels of PPARγ were significantly increased with incremental dosage of DHT treatment in rat granulosa cells, but decreased with bicalutamide treatment. Between-group comparisons were performed as indicated *P < 0.05, N = 3.

**Figure 6:**
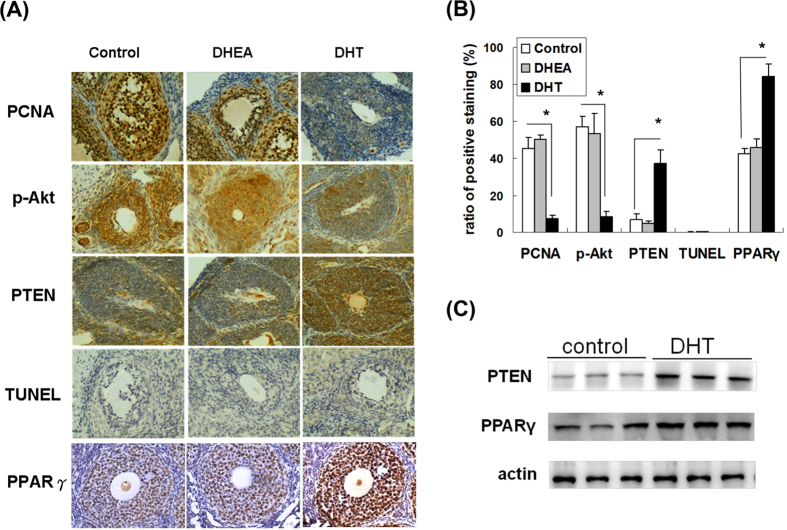
Expression of PCNA, p-Akt, PTEN, PPARγ and apoptosis marker in large antral follicles of placebo-, DHEA- and DHT-treated rats. (**A**) The expression of PCNA, p-Akt, PTEN, PPARγ and TUNEL assay. The representative immunohistochemical staining image of PCNA, p-Akt, PTEN, PPARγ and the TUNEL assay results using ovaries from placebo-, DHEA-, and DHT-treated rats are presented at 400x magnification. (**B**) Quantitative results of immunohistochemistry analysis. A total of 5 follicles per ovary were counted for analysis of granulosa cells with positive staining, and 3 ovaries per group and 1 ovary per rat were collected for analysis. The data are shown as means ± SD and represent comparisons with the control group *P   < 0.05. (**C**) Western blot analysis of PTEN and PPAR-γ proteins in ovarian tissue (N = 3/group). Each ovarian tissue was homogenized and extracted, the equal amount of protein extracts were subjected to immunoblotting as described in the materials and methods. Actin was used as an internal control.

Each experiment is repeated in more than three times, the data are expressed as the mean ± SEM or SD, unless otherwise indicated. All the statistical analyses were performed using the Statistical Analysis System (SAS version 9.3; SAS Institute Inc., Cary, NC, USA). *P* values less than 0.05 were considered statistically significant. The effects of DHT, DHEA or placebo on the rats’ weekly body weights and food intake amounts (Fig. 1) were analyzed using a repeated measures analysis of variance (ANOVA). The study results of Fig. 2, Table 1 and 2 were compared using ANOVA with Ducan post-hoc test for comparison among three groups. The Student’s *t* tests were applied for the between group comparison in Figs 3, 4, 5, 6.

## Results

### Body weight and food intake in androgenized rats

The weekly body weight changes and food consumption levels of the DHT-, DHEA- and placebo-treated groups are shown in Fig. 1A. The DHT-treated rat gained significantly more weight than the DHEA- and placebo-treated rats (*P* < 0.0001) by the end of the treatment period (Fig. 1A). In terms of the effect of androgens on food consumption, the average weekly food intake (*P* = 0.0012, Fig. 1B) and the cumulative food intake per rat during the entire study period (*P* = 0.0012) were significantly higher in the DHT-treated rats than in the other two groups, which contributed to pronounced weight gain of the DHT-treated rats.

### Disrupted estrous cyclicity and ovarian morphology

The vaginal smears revealed that the DHT-treated rats had significantly less frequent estrous cycles with longer estrous cycle intervals than did the placebo- and DHEA-treated rats, as shown in Fig. 2A. The ovaries of the placebo (control)- and DHEA-treated rats generally contained all the follicular stages in a single visual field (Fig. 2B), whereas the ovaries of the DHT-treated rats were more homogenous and contained a significantly higher number of large antral follicles (Fig. 2B,C). There were significantly less mature and ovulated follicles, including Graafian follicles and corpus lutea, indicating imminent and recent ovulations, in the DHT-treated rats compared with the placebo- and DHEA-treated rats (Fig. 2D).

### Body composition and metabolic and hormonal-related profiles

The weights of the hind limb muscles, subcutaneous fat depots, and visceral fat depots were significantly increased in the DHT-treated rats compared with the DHEA- and placebo-treated rats (Table 1). The DHT-treated rats had significantly lower circulating levels of testosterone, DHEAS and androstenedione but higher levels of LH and ALT compared with the placebo- and/or DHEA-treated rats, as shown in Table 2. The DHEA-treated rats had significantly higher levels of total cholesterol, HDL-C, and triglycerides, but lower adiponectin levels compared with the other 2 groups.

### Androgens inhibited FSH-induced granulosa cell proliferation *in vitro* by inducing cell cycle arrest at G2/M phase

To determine whether androgens affect the development of follicles through effect on granulosa cells, we then performed an *in vitro* primary culture of rat granulosa cells. We firstly evaluated the specificity of the antibody to FSHR of rat granulosa cells by comparing with human granulosa cells by immunoblotting (Fig. 3A, left panel), the result revealed the specificity of the antibody to FSHR of rat granulosa cells. The isolated rat granulosa cells were morphologically similar to human granulosa cells, and their purity was greater than 88%, as indicated by flow cytometric analysis of positive staining of FSH receptors compared with isotype controls (Fig. 3A, right panel). The rat granulosa cells were pre-treated with DHT, DHEA or insulin prior to FSH treatment to evaluate the effect of androgens on the granulosa cell proliferation (Fig. 3). The MTT assay performed to evaluate cell proliferation (shown in Fig. 3B) revealed that the FSH treatment significantly enhanced granulosa cell proliferation compared with no treatment. Pretreatment with DHT significantly suppressed the enhancement of cell proliferation by FSH in a dose-dependent manner. DHT was a stronger inhibitor of FSH-stimulated granulosa cell proliferation than was DHEA.

The MTT assay is considered as more an assay to measure the activity of the cellular metabolism than proliferation. We then further assessed the effect of DHT on cell cycle distribution in the FSH-treated granulosa cells using flow cytometry. As shown in Fig. 3C,D, the percentage of cells in G2/M phase decreased and cell proliferation increased after treatment with FSH in the granulosa cell culture. Compared with cells treated with FSH alone, the number of cells in G2/M phase remained unchanged after pretreatment with insulin but increased after treatment with DHT and FSH. The accumulation of G2/M-phase granulosa cells following DHT treatment implied that the suppressed FSH-stimulated granulosa cell proliferation caused by this treatment was due to cell cycle arrest at G2/M phase. Bicalutamide, a non-steroidal androgen receptor antagonist, was used to distinguish the effect of DHT via the androgen receptor or not. The results revealed that pretreatment of bicalutamide significantly reduced the DHT-caused G2/M phase granulosa cell accumulation. Such results demonstrated that the effect of DHT on inhibiting FSH-stimulated granulosa cell proliferation through G2/M cell cycle arrest might be a non-genomic effect via androgen receptors. The potential effect of insulin was also assessed in our study. Unlike DHT, the insulin treatment did not affect the FSH-stimulated cell cycle arrest (Fig. 3C,D). In addition, we also demonstrated that without the pre-treatment of FSH to stimulate granulosa cell proliferation, the DHT- or insulin- treatment alone or in combination did not affect the cell cycle distribution.

### DHT inhibited FSH-induced Akt phosphorylation but promoted PTEN expression in ovarian granulosa cells

The expression of p-Akt and PTEN has been reported to be involved in ovarian granulosa cell survival^22^,^23^ and follicular development^24^,^25^. In addition, the phosphatidylinositol 3-kinase (PI3K)/Akt signaling pathway is critically involved in FSH-mediated cell proliferation in granulosa cells^31^, and PTEN has been reported to be involved in cell cycle arrest and is a general negative regulator of cyclin D^32^. We therefore evaluated the effects of DHT on the expression of cyclins involved in the cell cycle and on FSH-induced Akt phosphorylation and PTEN expression in cultured granulosa cells (Fig. 4A,B). The results revealed that the expression levels of cyclin A, B, E, and D1 were all decreased under treatment with high-concentration DHT in granulosa cells. DHT, but not insulin, significantly suppressed the Akt phosphorylation induced by FSH treatment and restored PTEN expression. The increased expression of cyclin D1, representing the activation of the cell cycle and viability mediated by FSH, was also suppressed by DHT but not by insulin (Fig. 4B). The phosphorylation of PTEN on three residues (S380, T382, and T383) is likely to mediate the regulation of PTEN function^33^. We further determined the effect of DHEA and DHT on the PTEN and p-PTEN expression (Fig. 4C). The quantitative results revealed that DHT alone, but not DHEA, could apparently increase the expression of PTEN expression, as well as PTEN phosphorylation. As shown in Fig. 4D, DHT could significantly suppress the FSH-induced granulosa cell proliferation; however, the suppressive effect of DHT could be significantly reversed by PTEN siRNA treatment. These results indicate that DHT mediated PTEN expression plays a critical role in regulating FSH-induced granulosa cell proliferation.

### DHT promoted PTEN expression through enhancing PPARγ expression in ovarian granulosa cells

Previously, transcriptional factors as p53^34^, Egr-1^35^,^36^, and PPARγ^37^,^38^ have been reported to positively regulate the expression of PTEN. By using siRNA strategies, we then further investigated the potential contributing transcriptional factors that might be involved in the DHT promoted PTEN expression. As shown in Fig. 5A, the protein levels of p53, Egr-1 and PPARγ could be inhibited by their corresponding siRNA in rat granulosa cells. These results revealed that p53, Egr-1, PPARγ could be down-regulated by their siRNA in granulosa cells significantly. The increased mRNA expression of PTEN after DHT treatment was suppressed significantly by siRNAs of PPARγ but not by p53 or Egr-1 in granulosa cell culture (Fig. 5B). DHT treatment alone on the rat granulosa cells could apparently increase the expression levels of PPARγ both in protein (Fig. 5C) and mRNA (Fig. 5D) in a dose-dependent manner. Besides, bicalutamide significantly suppressed the DHT-induced PPARγ mRNA expression; it meant that the mechanism of action of DHT on PPARγ expression is via non-genomic and androgen receptor regulation (Fig. 5D). These results indicate that DHT-promoted PTEN expression is regulated by PPARγ expression in ovarian granulosa cells.

### Decreased cell proliferation and expression of phosphorylated Akt (p-Akt), and increased expressions of PPARγ and PTEN in granulosa cells from large antral follicles of DHT-treated rats

Since a short *in vitro* exposure to androgens is far from mimicking the long-term effects of the *in vivo* exposure, to address this question, we further compared the expression of p-Akt, PTEN, PPARγ, and PCNA in the large follicles of androgenized rat ovaries by immunohistochemical staining and quantified the numbers of positively stained cells (Fig. 6A,B). Compared with the placebo- and DHEA-treated rats, the expression of nuclear staining PCNA (indicating cell proliferation) in the large antral follicles was significantly lower in the DHT-treated rats. The cytoplasmic staining of phosphorylated Akt was suppressed; meanwhile, the cytoplasmic staining of PTEN and nuclear staining of PPARγ were enhanced in the granulosa cells of the large antral follicles from the DHT-treated rats. The TUNEL staining performed to detect apoptosis revealed no obvious differences in the apoptosis rates in the large antral follicles among the three groups. The results revealed significant reductions in cell proliferation and p-Akt expression, and an increase in PTEN and PPARγ expression in the granulosa cells of the large antral follicles from the DHT-treated rats. In addition, ovarian tissue (which contained granulosa cells from follicles) from rats that treated with vehicle (control) and DHT were used for the western blot analysis of PTEN and PPAR-γ proteins (Fig. 6C). The results revealed that DHT did enhance both PPARγ and PTEN expression. Taken together, our experiments indicate that DHT might suppress FSH-mediated granulosa cell proliferation by inhibiting FSH-activating Akt phosphorylation through enhancing PPARγ dependent PTEN expression and the resulting accumulation of large antral follicle (Supplementary Figure S1).

## Discussion

This study shows that the chronic exposure of prepubertal female rats to DHT, but not DHEA, induced significant increases in body weight and adiposity. Such findings confirm the results of previous studies suggesting that differential androgen levels in women with PCOS may lead to opposing phenotypes, including visceral obesity, insulin resistance and the risk of metabolic disorders^6^,^7^,^9^,^10^,^11^. In terms of the effects of androgens on female reproduction, the DHT-treated rats had higher numbers of large arrested follicles that resembled the polycystic ovaries observed in women with PCOS and may have been related to the reduced estrous cycles and lower numbers of mature and ovulated follicles in the rats. In this study, we showed that androgens not only cause obesity-related hyperinsulinemia but have independent effects on reproduction by increasing follicular arrest via the suppression of proliferation and the induction of cell cycle arrest in granulosa cells. These results may reflect regulation of p-Akt/PTEN expression by DHT.

DHT-treated female rats have previously^14^ been reported to have increase body weight, body fat, and insulin resistance. In the present study, we also found a higher body weight gain and body fat amount, but no evident insulin resistance in DHT-treated rat, in comparison to the other two groups. Exogenous DHEA has been reported to have anti-obesity effects, such as suppressing adipocyte proliferation and differentiation^39^, and reducing body weight and body fat in aged male rats and those fed a high-fat diet^40^,^41^, although the conclusions are controversial^42^. The discrepancies in the conclusions of the above-mentioned studies have been proposed to be due to differences in treatment length^42^,^43^. A longer period of DHEA treatment in old rats and those fed a high-fat diet produce more prominent reductions in body fat and weight companied by reduced energy intake compared with a control group^43^. However, little is known about the changes in body weight, body fat distribution and metabolism that follow chronic DHEA treatment in young female rats and those fed ordinary chow diets, and even less is known about its effects on reproductive function and hormonal profiles.

In this study, we found that chronic DHEA-treated female rats had significantly less body weight gain and food consumption levels, lower hind limb muscle weight and lower visceral and subcutaneous fat depots, and higher estrous cycles compared with the DHT-treated rats, but there were no significant differences compared with placebo-treated rats. The DHEA-treated rats exhibited higher blood levels of total cholesterol, HDL-C and triglycerides than the DHT- and placebo-treated rats. This result is likely due to the lipolytic effects of DHEA. DHT, the most potent androgen in the human body, is converted from testosterone by 5-alpha reductase in the hormone cascade. The downstream metabolites of DHEA include DHEAS, androstenedione and testosterone. The above reasons might explain the findings that the lower levels of androstenedione, DHEAS and testosterone, but higher levels of LH exhibited by the DHT-treated rats in comparison with DHEA-treated rats in this study. In addition, rat pituitary cell culture studies^44^,^45^,^46^ have reported that DHT treatment could decrease LH levels *in vitro* which might in turn lead to further decreased androgen production. The elevated hepatic enzyme in DHT-treated group was due to not only the effect of androgen, but also obesity. The association between elevated hepatic enzymes, obesity and androgen has been reported in women with PCOS^47^.

Chronic exposure to DHT, the most potent androgen receptor agonist, led to more pronounced and detrimental effects on reproduction and metabolism compared with chronic DHEA exposure. The different effects of DHT and DHEA might be due to their differential affinities for androgen receptors, their differential effects on PTEN expression and FSH-stimulated granulosa cell proliferation as shown in this study, their differential synergistic effects with other factors, such as FSH and IGF-1^48^,^49^, or the choice of young, female rats fed an ordinary chow diet as our experimental model. These differences may also be explained by the fact that DHEA is a pro-androgen and must be converted before it can exert effects. However, the length of exposure and the means of DHEA application may have also contributed to the discrepancies with the previous studies. Most of the previous studies of the effects of DHEA on rats have been brief (approximately 2-4 weeks), with DHEA administered inconsistently or under stressful conditions by daily oral feedings or intraperitoneal or subcutaneous daily injections^16^,^18^,^19^,^20^,^21^,^43^,^50^. Although there is no chronic DHEA-treated rat model available in the literatures, a recent study has reported a lack of PCOS phenotypes in a long-term (90 days) DHEA-treated mouse model^12^. In that study, the estrous cycle and body composition of DHEA-treated mouse showed no significant difference in comparison to the placebo-treated mouse^12^. Though the low dose of DHEA chosen in the mouse model might limit the interpretation, however, the results of that study were similar to those we found in our rat study.

Intraovarian hyperandrogenism is considered one of the most important determining factors of follicular arrest in both rodent models and women with PCOS^13^,^14^,^51^. Hyperandrogenemia in rat and mouse models have also reported to have decrease oocyte number, increase oocyte degeneration and impaired steroidogenesis in response to superovulation^52^,^53^,^54^. However, little is known about the contributing factors and mechanism underlying this process. As shown in the present study, DHT-treated rat and mouse models have increased numbers of large atretic follicles^13^,^14^, similar to the polycystic ovarian morphology of women with PCOS. We found that the large atretic antral follicles of the DHT-treated rats had prominently reduced ovarian granulosa cell proliferation and viability by PCNA assay; however, a TUNEL assay revealed that these results were not related to increased apoptosis. We then considered that androgens might affect ovarian follicular development through their effect on granulosa cell.

The PI3K-Akt signaling pathway is an important regulator of cell proliferation and survival that is widely involved in the pathogenesis of numerous types of cancer and disease^55^. PTEN, a tumor suppressor gene, is a negative regulator of PI3K and the cell cycle^32^. The regulation of Akt phosphorylation has been demonstrated to be involved in the granulosa cell survival^22^,^23^. Furthermore, the pathway of PTEN-PI3K-Akt has been studied in the regulation of dormant follicle activation^24^,^25^, and the inhibition of PTEN expression accompanied with Akt phosphorylation can promote follicular activation^24^,^56^. Using an *in vitro* rat granulosa cell culture, we proved that androgens, especially DHT, could strongly suppress FSH-stimulated granulosa cell proliferation by inducing cell cycle arrest at G2/M phase. DHT, unlike DHEA, could independently enhance the PTEN expression to promote the cell cycle arrest of granulosa cell by suppressing several cell cycle proteins, particularly cyclin D1. The subsequent inhibition of FSH-stimulated Akt phosphorylation might have also led to the suppression of granulosa cell proliferation and potentially to follicle arrest. The *in vivo* experiments performed in this study revealed that the large antral follicles of the DHT-treated rats had higher PTEN expression but reduced phosphorylated Akt and PCNA expression compared with the placebo and DHEA groups, in accordance with the above-mentioned findings.

Insulin has been reported to have an inhibitory effect on p-Akt by increasing PTEN expression in luteinized granulosa cells^28^. Hyperinsulinemia caused by DHT-related obesity may also affect follicular arrest. However, in this study, we could not replicate such effects of insulin using the primary granulosa cell culture isolated before luteinization.

PPARγ is an important transcriptional factor to regulate the metabolism, proliferation, inflammation and differentiation, and upregulates tumor suppressor genes as PTEN^37^,^38^. PPARγ is also involved in various metabolic disturbances^57^ as obesity^58^, cardiovascular disease, diabetes and PCOS^59^. Furthermore, it is also well known that PPARγ could down regulate the steroidogenesis in granulosa cell^60^. In this study, we firstly demonstrated that the DHT can directly upregulate the PTEN expression through enhancing the PPARγ expression levels, and then therefore, suppress the FSH-stimulated granulosa cell proliferation and affect the following ovarian follicle maturation. It has been reported that the DHT treatment impaired the steroidogenesis in granulosa cell, especially in response to FSH^53^. Though further study is still needed, it is reasonable to link the impaired steroidogenesis to the DHT-enhanced PPARγ expression. Excess androgen is considered detrimental to women’s health^47^,^61^ and reproduction^13^,^14^,^51^, as was also shown in this study; however, Sen *et al.* have demonstrated positive effects of a low dose of androgen on follicular development and fertility^62^. Therefore, in the future, it may be valuable to investigate the optimal androgen levels in the regulation of ovarian physiology.

In conclusion, we have demonstrated that chronic exposure to DHT or DHEA induces differential effects on body weight change, visceral and subcutaneous fat, and metabolic abnormalities in prepubertal female rats. Both the *in vitro* and the *in vivo* experiments demonstrated that the DHT treatment affected ovarian follicular maturation by altering granulosa cell proliferation and viability through the regulation of PPARγ and PTEN expression, phosphorylated Akt, and cell cycle arrest.

## Additional Information

**How to cite this article**: Chen, M.-J. *et al.* The effect of androgens on ovarian follicle maturation: Dihydrotestosterone suppress FSH-stimulated granulosa cell proliferation by upregulating PPARγ-dependent PTEN expression. *Sci. Rep.* **5**, 18319; doi: 10.1038/srep18319 (2015).

## Supplementary Material

Supplementary Information

## Figures and Tables

**Table 1:** Body compositions of placebo-, DHT- and DHEA-treated rats.

Weight (g)	Placebo rats	DHT rats	DHEA rats
Ovary	0.0924 ± 0.0270	0.0603 ± 0.0257	0.0804 ± 0.0145
Hind limb muscle	24.52 ± 1.23	28.61 ± 1.50^b^	22.72 ± 1.58^c^
Liver	10.69 ± 2.60	12.07 ± 0.49	12.41 ± 1.06
Visceral fat	9.70 ± 0.67^c^	12.02 ± 0.86^a,b^	7.99 ± 0.53^c^
Subcutaneous fat	6.70 ± 0.86^c^	9.62 ± 1.01^a,b^	5.77 ± 0.73^c^
Total body fat	16.40 ± 1.25^c^	21.64 ± 1.78^a,b^	13.76 ± 1.22^c^

The data are expressed as the mean ± SEM; n = 12–17 rats per group.

The weight of the ovary is expressed as the mean weight of both ovaries from each rat.

Total body fat was calculated as the sum of the visceral fat plus the subcutaneous fat.

^a^*P* < 0.05 vs. placebo group

^b^*P* < 0.05 vs. DHEA group

^c^*P* < 0.05 vs. DHT group

**Table 2:** Circulating hormonal and metabolic profiles of placebo-, DHT- and DHEA-treated rats.

	Placebo rats	DHT rats	DHEA rats
FSH (IU/L)	2.64 ± 0.63	3.25 ± 0.21	3.30 ± 0.28
LH (IU/L)	0.26 ± 0.06^b,c^	0.51 ± 0.04^ab^	0.02 ± 0.01^a,c^
E2 (pg/mL)	13.82 ± 2.37	6.55 ± 1.67	26.07 ± 12.79
P4 (ng/mL)	25.82 ± 3.95	13.20 ± 3.27	25.78 ± 6.10
Testosterone (ng/mL)	0.11 ± 0.02^b^	0.06 ± 0.02^b^	0.17 ± 0.02^a,c^
DHEAS (μg/dL)	2.08 ± 0.20^b^	1.13 ± 0.23^b^	3.20 ± 0.56^a,c^
Androstenedione	0.94 ± 0.71^c^	0.47 ± 0.23^a,b^	0.98 ± 0.10^c^
Glucose (mg/dL)	109.44 ± 8.23	114.37 ± 5.92	138.65 ± 21.17
Insulin (μg/L)	0.25 ± 0.04	0.48 ± 0.16	0.21 ± 0.04
Total cholesterol (mg/dL)	51.25 ± 2.93^b,c^	36.05 ± 3.51^a,b^	63.41 ± 4.07^a,c^
HDL-C (mg/dL)	22.67 ± 1.11^b,c^	16.43 ± 1.13^a,b^	27.43 ± 1.41^a,c^
LDL-C (mg/dL)	5.25 ± 0.52	5.11 ± 0.27	4.53 ± 0.37
Triglycerides (mg/dL)	54.12 ± 3.24^b^	61.21 ± 5.33^b^	83.53 ± 6.55^a,c^
AST (U/L)	142.75 ± 10.58	289.74 ± 65.11	226.41 ± 47.04
ALT (U/L)	40.37 ± 2.27^c^	61.68 ± 7.61^a^	47.00 ± 5.28
Adiponectin (μg/mL)	10.63 ± 0.91^b^	9.09 ± 0.60	8.26 ± 0.70^a^

The data are expressed as the mean ± SEM (n = 16–19 rats per group; FSH, LH, DHEAS, and androstenedione, n = 8–10 rats per group).

^a^*P* <0.05 vs. placebo group

^b^*P* < 0.05 vs. DHEA group

^c^*P* < 0.05 vs. DHT group.
