# Supplementary material for: The effect of androgens on ovarian follicle maturation: Dihydrotestosterone suppress FSH-stimulated granulosa cell proliferation by upregulating PPARγ-dependent PTEN expression
Source: Sci Rep. 2015 Dec 17;5:18319. doi: 10.1038/srep18319 (PMC4682139; doi:10.1038/srep18319)

Title: The effect of androgens on ovarian follicle maturation: Dihydrotestosterone suppress FSH-stimulated granulosa cell proliferation by upregulating PPARγ- dependent PTEN expression.

Author list: Mei-Jou Chen*, Chia-Hung Chou, Shee-Uan Chen, Wei-Shiung Yang, Yu-Shih Yang, and Hong-Nerng Ho

Figure legend:

Schematic for enhanced expression of PPARγ and PTEN by DHT and subsequent inhibition of FSH-mediated cell proliferation-dependent phosphorylated Akt signaling in granulosa cells.

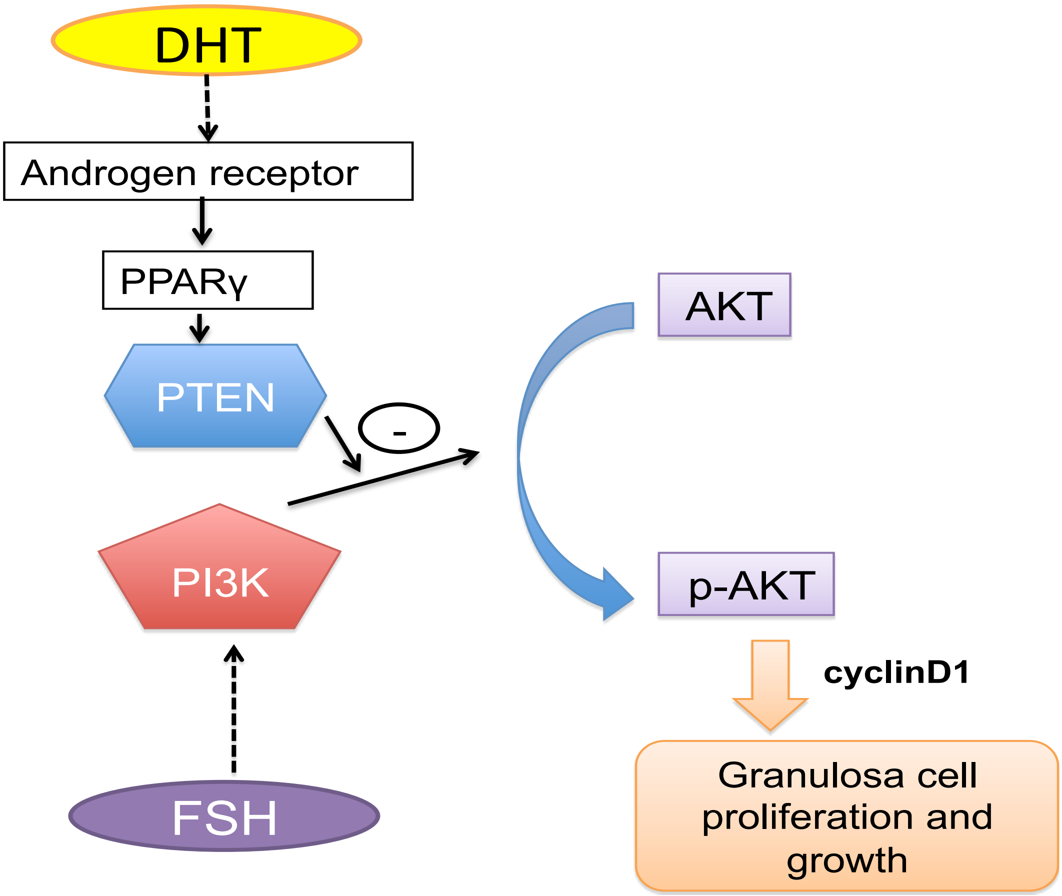

Supplement: Supplementary Information [file srep18319-s1.pdf]
